# Comparison of the image-derived radioactivity and blood-sample radioactivity for estimating the clinical indicators of the efficacy of boron neutron capture therapy (BNCT): 4-borono-2-^18^F-fluoro-phenylalanine (FBPA) PET study

**DOI:** 10.1186/s13550-016-0230-7

**Published:** 2016-10-18

**Authors:** Kayako Isohashi, Eku Shimosegawa, Sadahiro Naka, Yasukazu Kanai, Genki Horitsugi, Ikuko Mochida, Keiko Matsunaga, Tadashi Watabe, Hiroki Kato, Mitsuaki Tatsumi, Jun Hatazawa

**Affiliations:** 1 Department of Nuclear Medicine and Tracer Kinetics, Osaka University Graduate School of Medicine, 2-2, Yamadaoka, Suita City, Osaka 565-0871 Japan; 2 Department of Molecular Imaging in Medicine, Osaka University Graduate School of Medicine, Suita City, Osaka Japan; 3 Department of Radiology, Osaka University Graduate School of Medicine, Suita City, Osaka Japan; 4 Department of Immunology Frontier Research Center, Osaka University, Suita City, Osaka Japan

**Keywords:** FBPA, PET, BNCT, T/B ratio

## Abstract

**Background:**

In boron neutron capture therapy (BNCT), positron emission tomography (PET) with 4-borono-2-^18^F-fluoro-phenylalanine (FBPA) is the only method to estimate an accumulation of ^10^B to target tumor and surrounding normal tissue after administering ^10^B carrier of L-paraboronophenylalanine and to search the indication of BNCT for individual patient. Absolute concentration of ^10^B in tumor has been estimated by multiplying ^10^B concentration in blood during BNCT by tumor to blood radioactivity (T/B) ratio derived from FBPA PET. However, the method to measure blood radioactivity either by blood sampling or image data has not been standardized. We compared image-derived blood radioactivity of FBPA with blood sampling data and studied appropriate timing and location for measuring image-derived blood counts.

**Methods:**

We obtained 7 repeated whole-body PET scans in five healthy subjects. Arterialized venous blood samples were obtained from the antecubital vein, heated in a heating blanket. Time-activity curves (TACs) of image-derived blood radioactivity were obtained using volumes of interest (VOIs) over ascending aorta, aortic arch, pulmonary artery, left and right ventricles, inferior vena cava, and abdominal aorta. Image-derived blood radioactivity was compared with those measured by blood sampling data in each location.

**Results:**

Both the TACs of blood sampling radioactivity in each subject, and the TACs of image-derived blood radioactivity showed a peak within 5 min after the tracer injection, and promptly decreased soon thereafter. Linear relationship was found between blood sampling radioactivity and image-derived blood radioactivity in all the VOIs at any timing of data sampling (*p* < 0.001). Image-derived radioactivity measured in the left and right ventricles 30 min after injection showed high correlation with blood radioactivity. Image-derived blood radioactivity was lower than blood sampling radioactivity data by 20 %. Reduction of blood radioactivity of FBPA in left ventricle after 30 min of FBPA injection was minimal.

**Conclusion:**

We conclude that the image-derived T/B ratio can be reliably used by setting the VOI on the left ventricle at 30 min after FBPA administration and correcting for underestimation due to partial volume effect and reduction of FBPA blood radioactivity.

## Background

Boron neutron capture therapy (BNCT) requires selective delivery of ^10^B-containing drug to the tumor and irradiation of the tumor with neutrons. Nuclear reaction yields high-linear-energy-transfer α particles and recoiling ^7^Li nuclei in the body [1, 2]. BNCT was effective in patients with inoperable, locally advanced brain tumors, head and neck cancers and melanomas, even in those with tumor recurrence at previously irradiated sites [3–5]. Successful application of BNCT requires selective delivery of ^10^B to the tumor [1, 6]. In the BNCT practice, the ^10^B carrier used has been L-paraboronophenylalanine labeled with ^10^B and conjugated with fructose (BPA-fr) [7–9]. We previously reported that 4-borono-2-^18^F-fluoro-phenylalanine (FBPA) PET could predict BPA-fr accumulation in the tumors transplanted to rats [9]. Imahori et al. compared the ^10^B accumulation in tumors estimated by the rate constants measured by means of FBPA PET with that in surgically resected specimens of high-grade gliomas using the prompt gamma method and showed similar concentration of ^10^B in the tumor [10].

In the current BNCT, FBPA PET has been performed to predict the accumulation of ^10^B in the tumors and normal tissues [6, 8, 10–17]. Absolute concentration of ^10^B in the tumor has been estimated by multiplying ^10^B concentration in the blood during neutron irradiation by tumor-to-blood radioactivity (T/B) ratio derived from FBPA PET. Although blood radioactivity in T/B ratio has been measured by taking venous blood samples during FBPA PET, the method to measure blood radioactivity either by blood sampling or image data has not been standardized yet [4, 10, 12–14, 18]. We compared image-derived blood radioactivity of FBPA with blood sampling data and studied appropriate timing and location for measuring image-derived blood counts in order to estimate T/B ratio in the tumor.

## Methods

### Subjects

This study was performed with the approval of the institutional ethics committee for clinical research of Osaka University. Written informed consent was obtained from each subject, after they were provided a detailed explanation about the procedures of the study.

A total of 5 healthy volunteers (three males and two females) participated in the present study after they received a detailed explanation about the radiotracer drug, the purpose and contents of the study. The mean age of the 5 healthy volunteers was 34 years (range, 21 to 56), and the mean height and weight were 167 cm (range, 160 to 174 cm) and 61 kg (range, 48 to 66 kg), and the mean body mass index were 21.7 ± 1.8 (range, 18.8 to 24.5), respectively. None of the healthy volunteers had any prior history of any major medical illness.

### Preparation of radiotracers

FBPA was prepared by a previously described method, with several modifications [7, 9, 19]. The F-1 synthesizer (Sumitomo Heavy Industries, Tokyo, Japan) was used. ^18^F-acetylhypofluorite in Ne was bubbled at a flow rate of 600 mL/min at room temperature into 5 mL of trifluoroacetic acid containing 30 mg of 4-borono-L-phenylalanine (Matrix SCIENTIFIC, COLUBIA, SC, USA). Trifluoroacetic acid was removed by passing N_2_ under reduced pressure at a flow rate of 200 mL/min. As in previous studies, the residue was also dissolved in 3 mL of water containing 0.1 % acetic acid, and the solution was applied to a high-performance liquid chromatography column, YMC-Pack ODS-A S-5 (20 × 150 mm; YMC, Kyoto, Japan), with water for injection containing 0.1 % acetic acid as the mobile phase, flow rate of 10 mL/min, ultraviolet detection at 280 nm, and a radioactivity detector. The FBPA fraction (retention time = 19 to 21 min) was collected by adding 25 % ascorbic acid injection and 10 % sodium chloride injection. The specific activity of FBPA was 49.7 ± 17.3 GBq/mmol as determined by HPLC. The radiochemical purity of FBPA was >98 %.

### PET image acquisition

All the healthy volunteers were instructed to fast for at least 4 h before the radiotracer injection [20, 21]. Before injecting each radiotracer, the volunteers were asked to void their bladders. Whole-body PET scans were performed using Eminence SOPHIA SET-3000 BCT/X (Shimadzu Co, Kyoto, Japan) in the three-dimensional acquisition mode. Transmission data using a rotating ^137^Cs point source for attenuation correction were acquired. The amount of injected activity was measured in a dose calibrator (196 ± 16 MBq). Whole-body emission scans were initiated simultaneously with injection of the radiotracer into the antecubital vein at the rate of 5.2 mL/min. Seven repeated whole-body PET scans from the parietal crown to the groin were performed in each of the five healthy volunteers. The data consisted of seven scans with an acquisition time of 455 sec, and an interval between scans of 45 sec. Radioactivity decay during the PET scan was corrected for the FBPA injection time. All the images were reconstructed using Dynamic Row-Action Maximum Likelihood Algorithm (DRAMA) with an image matrix of 128 × 128, resulting in a voxel size of 4.0 × 4.0 × 3.25 mm^3^ [21]. The axial field of view was 26 cm. After the PET scanning, whole-body CT (80 kV, 135 mAs) was performed for image fusion.

### Time-activity curves (TACs) of blood sampling radioactivity

Before and during the PET study, nine arterialized venous blood samplings were obtained: at background, 30 sec, and 1, 3, 5, 10, 20, 30 and 50 min after the tracer injection from the antecubital vein contralateral to the intravenous FBPA injection side, heated in a heating blanket. The radioactivity (cps/g) of the whole blood was measured in a cross-calibrated well-type scintillation counter (Shimadzu Co, Kyoto, Japan), and fixed in attenuation correction to the FBPA injection time.

### TACs of PET image-derived blood radioactivity

The radioactivity in various blood pools was obtained from reconstructed PET images by averaging the activities in each blood pool, since the radioactivity distribution within a blood pool can be considered to be uniform [22]. TACs of the image-derived blood radioactivity were obtained using volumes of interest (VOIs) over the ascending aorta, aortic arch, pulmonary artery, left and right ventricles, inferior vena cava and abdominal aorta. Spherical VOIs with a diameter of 10 mm were set on these blood pools of the PET images referring to the individual CT images. VOIs were drawn on 3 cross-sections of the tomographic images. Data were obtained for each of the organs using PMOD software, ver.3.6. TACs were created using the average values of five VOIs consisting of regions of interest drawn over each blood-pool area.

### FBPA metabolite analysis

The proportion of ^18^F radioactivity in the plasma present as FBPA was measured in 5 healthy subjects. Blood samples were collected at 20 min and 50 min after the FBPA injection from the antecubital vein contralateral to the intravenous FBPA injection side. Radiochemical purity was analyzed by HPLC [10].

### Data analysis

We verified whether the image-derived blood radioactivity was correlated with the blood sampling radioactivity in the healthy subjects. TACs of the blood sampling radioactivity were compared with those of the image-derived blood radioactivity. The TAC of the image-derived radioactivity within each blood pool was calculated as the average of five radioactivities (cps/g), respectively. The blood sampling radioactivity at 3, 20, 30 and 50 min after the tracer injection and the image-derived blood radioactivity at each VOI (ascending aorta, aortic arch, pulmonary artery, left and right ventricles, inferior vena cava, and abdominal aorta) in mid-scan time at 3.8, 20.5, 28.8 and 53 min after the tracer injection were compared.

The magnitude of underestimation of blood radioactivity counts was examined. The timing of the minimal reduction of image-derived blood FBPA radioactivity was examined from TAC, and reduction of blood radioactivity of FBPA in each blood pool was calculated and compared, respectively.

### Statistical analysis

The relationships between the blood sampling radioactivity levels and the radioactivity level within each blood pools on the PET images were analyzed by with linear regression and Spearman’s correlation tests. The correlation coefficient was also estimated as a statistic to express the comparability of these values.

The proportion of blood sampling radioactivity and image-derived radioactivity and that of reduction rate was described as the mean ± SD. Mean values were compared using a one-way analysis of variance. In all the statistical analyses, significance was defined as a *P* value of less than 0.05. All the statistical analyses were performed with StatMate IV (ATMS Co., Ltd., Tokyo, Japan).

## Results

**Fig. 1:**
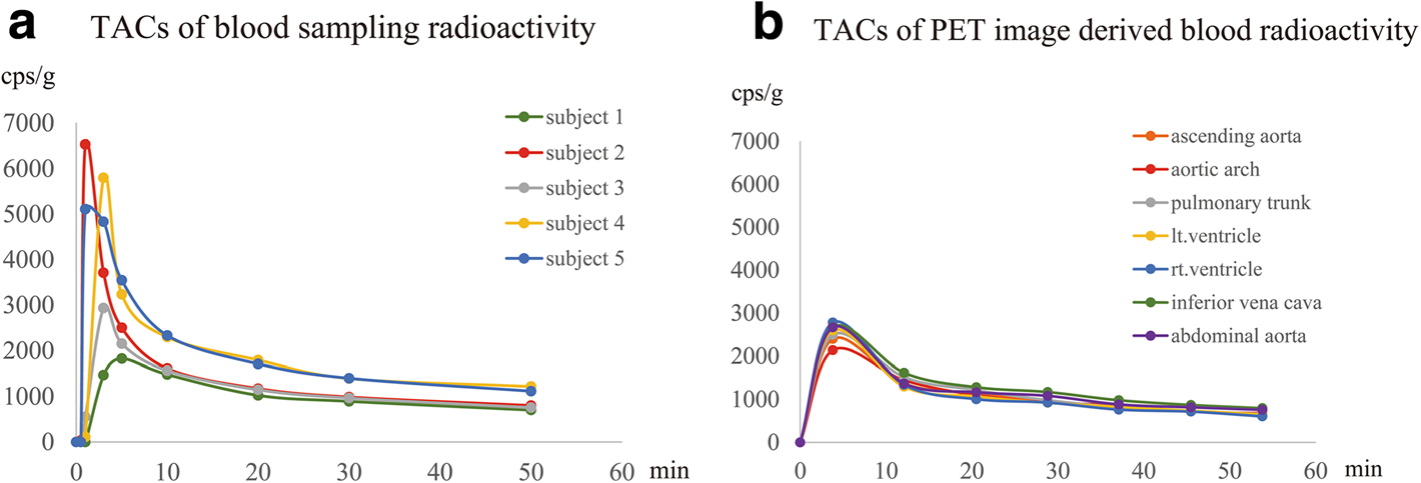
Time–activity curves (TACs). TAC of blood sampling radioactivity in 5 healthy subjects (**a**) and TAC of PET image-derived blood radioactivity: average values from 5 healthy subjects (**b**). Both the TACs showed a peak within five min after the FBPA injection, gradually approaching a constant level at 20 min after the injection

**Fig. 2:**
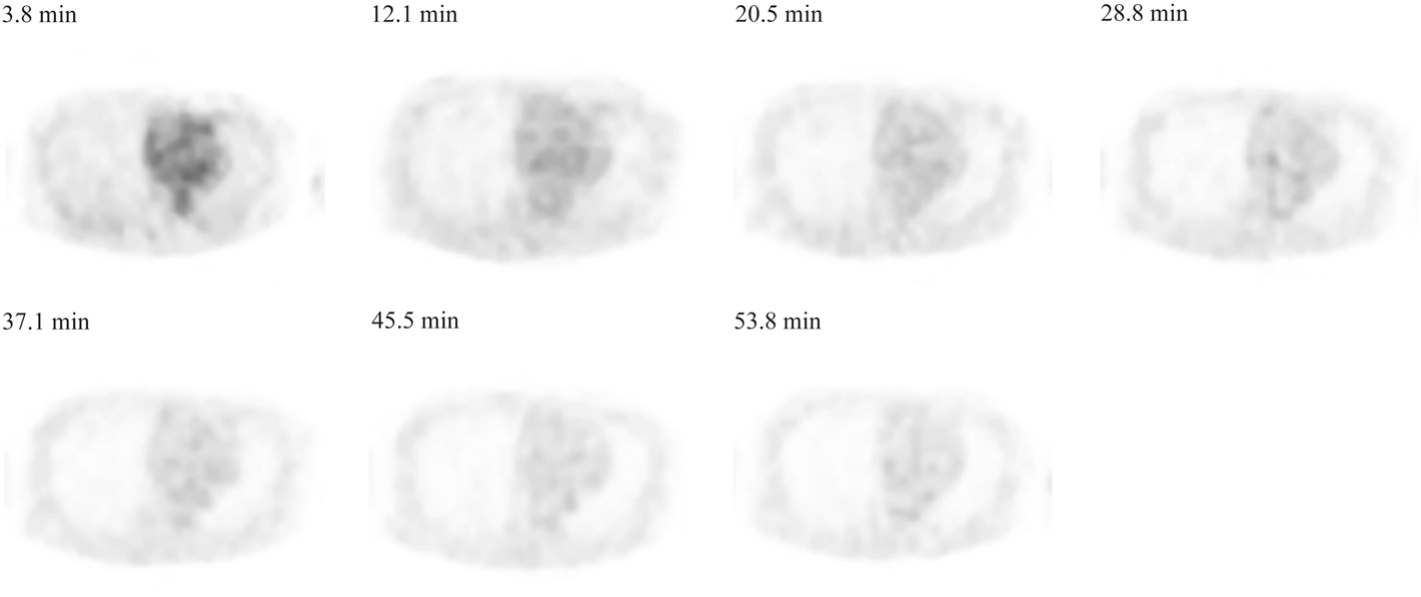
Time-course of the PET image-derived blood radioactivity. Representative decay-corrected axial images of the left ventricle in a 56-year-old healthy woman after injection of FBPA. High accumulation in the left ventricle was observed soon after the FBPA injection, to promptly decrease soon thereafter and gradually approach a constant level at 20 min later after the tracer injection

The TACs of the blood sampling radioactivity showed a peak within five min after injection in each subject, promptly decreasing thereafter (Fig. 1a). The FBPA radioactivity gradually approached a constant level at 20 min after the injection. The peak of the image-derived blood radioactivity was found in the first image in all blood pool structures in each subject, and gradually approaching a constant level at 20 min after the injection (Fig. 1b). Representative PET tomographic images of the blood pools are shown in Fig. 2.

**Fig. 3:**
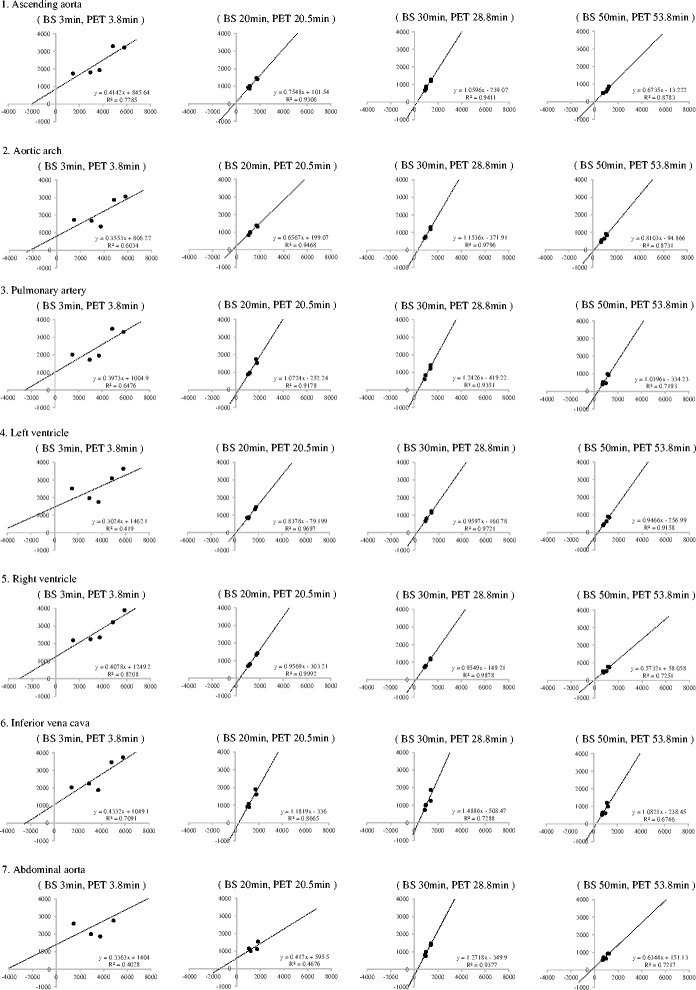
Correlation between blood sampling radioactivity and image-derived radioactivity in each blood pool. The blood samplings radioactivity (BS) at 3, 20, 30 and 50 min after the tracer injection and the image-derived blood radioactivity (PET) at each site (ascending aorta, aortic arch, pulmonary artery, left and right ventricle, inferior vena cava and abdominal aorta) in mid-scan time at 3.8, 20.5, 28.8 and 53 min after the injection were compared. BS is plotted on the horizontal axis, and PET is plotted on the vertical axis. Both units are in cps/g. A linear relationship was found between blood sampling radioactivity and PET image-derived radioactivity in each blood pool (*p* < 0.001). Strong correlations were observed between these parameters at 20 min after FBPA administration. The image-derived radioactivity measured in the left ventricle and right ventricle 30 min after injection showed high correlation (0.97 and 0.99, respectively), slope close to be 1.0 (0.96 and 0.95, respectively), and intercept (-161 and -149) with blood radioactivity

A linear relationship was found between the blood sampling radioactivity and image-derived radioactivity in each VOI (Spearman’s, *p* < 0.001) (Fig. 3-1, 3-2). The image-derived radioactivity measured in the left ventricle and right ventricle at 30 min after injection showed high correlation (0.97 and 0.99, respectively), slope close to be 1.0 (0.96 and 0.95, respectively), and intercept (-161 and -149) with blood radioactivity.

Table 1 summarized a proportion of image-derived radioactivity to blood sampling radioactivity in each sampling time. Mean proportion of image derived value was 80 % of blood sampling radioactivity. Although there was no statistically significant difference, the magnitude of underestimation was changed depending on the sampling time. In most of the blood pool, the underestimation became larger. In contrast, the underestimation of the image-derived radioactivity in the inferior vena cava and abdominal aorta were relatively small as compared to other sites at 30 min after the injection.

**Table 1:** Compaison of the proportion of blood sampling radioactivity (BS) and image-derived radioactivity (PET)

	Ascending aorta	Aortic arch	Pulmonary artery	Left ventricle	Right ventricle	Inferior vena cava	Abdominal aorta
PET 3.8 min/BS 3 min (%)	71 ± 24	65 ± 28	76 ± 32	83 ± 45	84 ± 32	80 ± 30	85 ± 47
PET 20.5 min/BS 20 min (%)	83 ± 6	81 ± 4	88 ± 7	78 ± 4	72 ± 4	92 ± 11	88 ± 15
PET 28.8 min/BS 30 min (%)	84 ± 7	81 ± 7	86 ± 10	81 ± 4	82 ± 3	102 ± 18	95 ± 9
PET 53.8 min/BS 50 min (%)	66 ± 5	71 ± 6	68 ± 15	67 ± 8	64 ± 8	82 ± 15	80 ± 10

Data are mean ± SD

Table 2 showed a reduction rate per min (%/min) of image-derived FBPA radioactivity in each blood pool during the period between 30 to 50 min after injection. The minimal reduction rate of image-derived blood FBPA radioactivity was found in the left ventricle, but no significant difference was found. Mean reduction rate of blood sampling radioactivity was 0.95 ± 0.17 %/min.

**Table 2:** Comparison of the proportion of the reduction rate of image-derived radioactivity

	Ascending aorta	Aortic arch	Pulmonary artery	Left ventricle	Right ventricle	Inferior vena cava	Abdominal aorta
reduction rate (%)/min	1.32 ± 0.29	1.05 ± 0.30	1.37 ± 0.28	1.23 ± 0.30	1.37 ± 0.23	1.27 ± 0.27	1.13 ± 0.48

Data are mean ± SD

In Table 3, the fraction of un-metabolized FBPA in plasma at 20 min and 50 min after injection was shown in each subject. Mean fraction of un-metabolized FBPA at 20 min and 50 min was 97.68 ± 1.57 % and 96.03 ± 1.64 %, respectively.

**Table 3:** FBPA metabolite measurements

Subject number	The proportion of unchanged FBPA after 20 min (%)	The proportion of unchanged FBPA after 50 min (%)
1	97.63	95.18
2	98.15	95.23
3	94.72	93.87
4	99.17	98.17
5	98.71	97.72

## Discussion

In the BNCT practice, the radioactivity of tumor (T) and surrounding normal tissue (N) has been measured by the FBPA PET, and the blood radioactivity (B) has been measured by means of well-counter. The well-counter radioactivity was usually converted to the PET count using predetermined cross calibration factor (CCF). The CCF is changeable depending on the performance of the PET scanner and sensitivity of well-counter and one of the source of errors for the accurate estimation of T/B ratio and N/B ratio. Therefore, we assumed that the radioactivity measurements for T, N, and B in the same equipment will make the T/B and N/B estimation more reliable and easier.

When the image-derived blood count of FBPA is used for the calculation of T/B ratio, the radioactivity in the tumor and that in the blood should be measured at the same time. However, when brain tumors and head and neck tumors are studied, the time discrepancy of PET measurement between the tumors and blood pool in the chest is inevitable. In this situation, the blood radioactivity at the time of brain scan and that at the time of body scan should be almost equal. In the present study, the reduction of blood radioactivity of FBPA after 30 min of injection was less than 0.58 %/min, consistent with previous study [14]. Therefore, the PET imaging for tumor and blood pool should be performed 30 min or later after injection in order to minimize the discrepancy of the blood radioactivity by the difference of scan time. The best location of VOI setting was left ventricle because of minimal reduction of blood FBPA counts.

The image-derived radioactivity measured in the left ventricle and right ventricle 30 min after injection showed high correlation (0.97 and 0.99, respectively), slope close to be 1.0 (0.96 and 0.95, respectively), and intercept (-161 and -149) with blood radioactivity. In other blood pools and other timing at 30 min or later after injection, either of correlation coefficient, slope or intercept was not sufficient. In the aortic arch at 30 min after injection, the correlation coefficient, slope, and intercept was 0.98, 1.15, and -372, respectively. Therefore, either left or right ventricle was appropriate for setting VOI.

The present study indicated that image-derived blood radioactivity underestimated blood-sampling radioactivity. When left ventricle and right ventricle were used for the image-derived sampling, image-derived blood activity was 81 % and 82 % of blood sampling radioactivity, respectively. Possible reasons for this underestimation were partial volume effect due to the limited spatial resolution of the current scanner, beat of the heart, and respiratory movement [23]. Another inaccuracy of left ventricular image-derived data might be due to spillover of FBPA accumulated in the myocardium. As shown in Fig. 2, however, physiological accumulation of FBPA in the myocardium is low. In contrast, image-derived blood counts in the inferior vena cava (102 %) and abdominal aorta (95 %) were higher than those in other regions. This might be due to the spillover from liver, kidney and urinary system where physiological accumulation or excretion of FBPA were found [18].

^10^B concentration in the tumor during neutron irradiation has been estimated by multiplying mean ^10^B concentration in the blood measured just before and after irradiation by the T/B ratio of FBPA PET [4]. Since the image-derived blood counts of FBPA were 81 % and 82 % of blood-sampling counts of left and right ventricles, respectively, image-derived T/B ratio need a correction for the underestimation.

The present study showed that mean fraction of un-metabolized FBPA in plasma at 20 min and 50 min after injection was 97.68 % and 96.03 %, respectively. Imahori et al. also reported negligibly small fraction of FBPA metabolites in plasma and suggested no need of correction for metabolites fraction [11]. It remained unknown whether the FBPA metabolites were trapped by tumors and normal tissues.

There were limitations of this study. Firstly, although we collected blood samples for measuring the radioactivity levels during the scan, the blood sampling time was not consistent with the scan time of each blood pool. The blood sampling radioactivity measured to the nearest time and the radioactivity of the blood pool on the PET images were compared. Secondly, regarding the underestimation of tumor radioactivity (T) due to the partial volume effect of the current PET scanner, it has been recommended to use maximal value of tumor radioactivity within the voxel of interest. We expect the improvement of spatial resolution and sensitivity of the present PET scanner and the development of, appropriate image reconstruction algorithm and image analysis software may be better for spatial resolution and sensitivity.

## Conclusions

Image-derived T/B ratios can be a substitute for blood sampling-based T/B ratios. However, the value of image-derived blood radioactivity represents an underestimate in comparison with the value of blood sampling-based blood radioactivity. The best location of VOI setting was left ventricle because of minimal reduction of blood FBPA counts. We conclude that the image-derived T/B ratio can be reliably used by setting the VOI on the left ventricle at 30 min after FBPA administration and correcting for underestimation due to partial volume effect and reduction of FBPA blood radioactivity.
